# Assessing the acceptability of photographs and medical illustrations in Buruli ulcer health communication among health providers, community volunteers and community members in endemic districts of Ghana

**DOI:** 10.1371/journal.pntd.0014275

**Published:** 2026-04-22

**Authors:** Ruth Dede Tuwor, Joanna Butler, Samuel Tetteh, Emmanuel Boakye Boateng Okyere, Miriam Gborglah, Kabiru Mohammed Abass, Abigail Agbanyo, Bernadette Agbavor, Caroline Erolin, Roderick Hay, Richard Odame Phillips, Yaw Ampem Amoako, Rachel E. Simmonds

**Affiliations:** 1 Kumasi Centre for Collaborative Research in Tropical Medicine, Kwame Nkrumah University of Science and Technology, Kumasi, Ghana; 2 Discipline of Microbes, Infection & Immunity, School of Biosciences, Faculty of Health and Medical Sciences, University of Surrey, Surrey, United Kingdom; 3 Medical Artist Ltd, Haslemere, Surrey, United Kingdom; 4 Agogo Presbyterian Hospital, Agogo, Ghana; 5 Centre for Anatomy and Human Identification, University of Dundee, Medical Sciences Institute, Dundee, United Kingdom; 6 King’s College, London, United Kingdom; 7 School of Medical Sciences, Kwame Nkrumah University of Science and Technology, Kumasi, Ghana; Johns Hopkins University, UNITED STATES OF AMERICA

## Abstract

**Background:**

The control of Buruli ulcer (BU) relies on early case detection to improve disease outcomes. While lesion photographs are currently used in health communication materials, they have many drawbacks. Medical illustrations, though proven effective in many contexts, remain unutilised for skin NTDs. We therefore aimed to assess their acceptability in health communication materials in BU-endemic districts of Ghana.

**Methodology/principal findings:**

We conducted eleven focus group discussions, using a guide developed from the PICO framework and the Theoretical Framework of Acceptability (TFA). Participants were shown photographs and clinically approved medical illustrations of different presentations of BU. All participant groups confirmed that images of BU play a key role in health communication. However, photographs of the disease may frighten the viewer, either deliberately or not, especially when they show advanced stages of the BU resulting in revulsion and anxiety. All agreed that illustrations were acceptable and do not evoke the same negative emotional response, whilst still providing accurate information for detection. Health providers expressed the view that the psychological distress associated with real-life images are beneficial for prompt healthcare-seeking; however, the views of community members contradicted this. Health providers and CBSVs currently manage such negative reactions through a variety of methods. However, these strategies do not always work as some viewers shy away from the materials.

**Conclusion:**

Medical illustrations have a potentially important role to play in BU health messaging. As well as their role in communicating the characteristic features of the disease without distracting elements or ethical issues, they are also particularly well-suited to depict BU in the later stages to produce health communication materials that are sensitive to the emotional states of viewers. We encourage health communicators to place materials within appropriate social and cultural contexts, to promote their effectiveness and uptake of the health messaging.

## Author summary

Buruli ulcer (BU) is a disease that affects many people in rural communities in endemic countries. Prevention strategies are highly dependent on detecting cases early and beginning timely appropriate treatment. To achieve this, health education for stakeholders such as community members and health providers is central. In this study, we held discussions with health providers, community health volunteers and community members from affected communities in selected BU endemic districts in Ghana. We sought to explore the effects and acceptability of the real-life images used in current health messaging materials versus medical illustrations. Health providers, community volunteers and community members noted that images play a key role in recognising and learning about the disease. However, the currently used photographs can also cause fear, anxiety and, ultimately, avoidance. All participant groups noted that medical illustrations will likely play an invaluable complementary role for engaging sensitive audiences when portraying the advanced stages of the disease and within community contexts. Community members particularly valued medical illustrations for emotional comfort. Our findings show that medical illustrations can complement photographs to make BU health messaging both effective and acceptable for people. We recommend that BU health communicators consider carefully when and where illustrations might be more effective than photographs and incorporate it into messaging to help attaining the global target of BU control.

## Introduction

Buruli ulcer (BU), a debilitating skin Neglected Tropical Disease (skin-NTD) is caused by infection with the toxin producing *Mycobacterium ulcerans*. BU is common in rural areas of West Africa but currently, cases are on the rise in parts of Australia [1–4]. Typically, BU presents as a painless nodule or plaque, characterised by a raised, firm and discoloured portion of the skin or non-pitting oedema that can affect the whole limb or surround an ulcer. Subsequently, the lesion enlarges and ulcerates, with the ulcer having a characteristic necrotic base. Current treatment is with combination rifampicin and clarithromycin complemented with good wound care that comprises cleansing with normal saline, covering with vaseline gauze, and short-stretch bandaging to reduce surrounding oedema.

The control of BU relies on early case detection by community-based health care workers and initiation of early appropriate antibiotic therapy. Late stages (category III) of the disease may be associated with poor outcomes including delayed healing or healing with disfigurement and disability, especially when lesions are located close to joints. The World Health Organisation (WHO) has developed a roadmap for tackling neglected tropical diseases and, for BU, this envisions the proportion of category III BU - severe late stage lesions - to decrease to less than 10% worldwide by 2030 [5]. To achieve this, active community education and awareness campaigns are essential as the local understanding and practices of the disease can either aid or frustrate control and elimination efforts.

Several NTD actors and stakeholders, including the WHO, have fostered education and promoted knowledge of disease presentations and management for affected communities and health workers by providing printed (such as posters, booklets, leaflets, brochures) and/or digital (such as Apps, videos, PowerPoint slides) educational materials showing lesion images [6, 7]. Simple pictorial guides using photographs are considered helpful for training health providers and village health volunteers [8]. In developing mobile applications to aid the diagnosis of skin diseases in resource-poor settings, the inclusion of pictorial images or graphics of disease signs and symptoms, is considered crucial for improving disease diagnosis [9,10].

Although it is acknowledged that health communication is an important aspect of control measures for achieving skin NTD elimination targets [5], not much attention is given to well-developed and theorised approaches to delivering culturally sensitive, effective health messaging to endemic communities. Materials and tools are often developed using a top-down approach, and communities often have little or no role to play except in receiving such materials [11–13]. However, effective health communication is achieved when people are not just recipients of health messaging but are also involved in both the co-creation and evaluation activities to guarantee the effectiveness and contextual validity of the material.

In community outreach activities for BU, as with other skin NTDs, the images used in health communication materials have been real-life imagery (such as photographs or videos) of people with the disease. These provide a visual window into the condition of affected people, allowing the viewer to grasp the associated preventive and curative messaging. Photographs are frequently included in posters, videos, and leaflets to increase community knowledge by showing the signs and symptoms, treatments available and the need to seek early and appropriate treatment [14,15]. However, photographs can present various technical issues, having been taken in a clinical environment. These include variations in lighting (and therefore producing shadows), inconsistent views (either close-up or long distance), distracting backgrounds (clothing, surroundings, other people), or camera angles that make it hard to determine the lesion site. In addition, where photographs were taken using flash, the bright light exaggerates the appearance of the condition due to the increase in contrast. Here, red colours in particular can look more intense leading to images that appear less pleasant than under natural light.

There are also ethical issues, relating to patient consent and confidentiality when considering their reuse in publicly available material. It is also noted that the emotional reaction to photographs may either increase or decrease the target behaviours, depending on the emotional response elicited in viewers [16,17]. On the other hand, medical illustrations can convey complex medical and anatomical information to capture the characteristic features of a disease from many different cases and bring them together into a single image that allows the condition to be recognised in once glance. They can be ‘lit’ consistently, in the absence of distracting backgrounds, or with the addition of appropriate backgrounds depending on the audience. Using techniques such as “call-outs” the pathology can be shown at low and high magnification, and there are no ethical issues relating to consent. The tradition of medical illustration to communicate human anatomy, pathology and health-related subject matter has stood the test of time as a visual communication method used in educational texts at all levels. Skin NTDs, and particularly BU, remain very under-represented in terms of available images, especially those that have been approved by clinicians familiar with the disease presentation. Filling this gap would have great potential to be utilised as training materials and in the community. However, there remains an underexplored domain on the perceptions, cultural appropriateness and visual appeal of these pictorial materials, even though this may affect the reception that is given to the education that these materials are intended to provide [11]. We recently found that educational materials featuring photographs of BU may be less effective in some contexts, as it was reported that community members shied away from them when shown directly, or when they appeared in videos, PowerPoints or posters for example describing them as ‘unpleasant’ [18]. Therefore, medical illustrations of BU might have a dual role, both to enhance recognition of the disease, and to change the way that the viewer engages with the image. Whereas photographs can induce negative emotional responses, illustrations instead can provoke curiosity, logic and analysis. This is well-accepted for medical illustrations of a wide range of diseases and/or medical procedures, for instance illustrated communication materials have been noted to contribute to a reduction in anxiety levels and increase patient satisfaction and comfort levels in some conditions [19,20]. Despite this, medical illustrations are currently unutilised in the context of skin NTDs. We therefore aimed to assess the acceptability of medical illustrations of BU in health communication materials by health providers and community-based surveillance volunteers and community members in BU-endemic districts of Ghana.

## Methods

### Ethics statement

Ethical approval was obtained from the Committee on Human Research Publication and Ethics (CHRPE) at the Kwame Nkrumah University of Science and Technology (approval number: CHRPE/AP/544/24). Full disclosure and explanation of the study procedures were provided to participants in their preferred language (English or the local language, Twi). Written informed consent was obtained from all study participants. All study staff received training on the study aims and appropriate data collection strategies. All the study processes were guided by the principles guiding research in human subjects as set out in the Declaration of Helsinki [21].

### Study setting

This study was conducted in the Asante Akyem North, Ahafo Ano North, and Atwima Mponua districts of the Ashanti region of Ghana. This region is located in the middle belt, and shares boundaries with the Eastern, Western, Central and the Bono, Bono East, and Ahafo regions. The Ashanti region has a total population of 5,440,463 and occupies an approximate area of 24,389 square kilometres [22].

The districts were selected because they are known to be endemic for BU disease. The Kumasi Centre for Collaborative Research (KCCR) collaborates with the Ghana Health Service to operate clinics that provide care for individuals with skin NTDs in these districts [2,23]. Some activities organised routinely within the districts include health communication, case search and management activities. Many of these activities have been conducted with the assistance of educational materials with photographs in the form of posters, booklets, videos, photo books, and other forms of educational material of BU lesions, featuring both the pre-ulcerated and ulcerated states of the disease [24–27].

### Medical illustrations of BU

The illustrations of BU for this study were created using a range of scientific principles to ensure clinical accuracy and to have the greatest chance of supporting the communication of BU in various settings. The illustration method was developed using established theories and design principles to create a systematic approach to illustration development [28]. These include Gestalt principles of visual perception (that the composition of images or grouping of a set of images can enhance the utility of a design) [29], the ‘use of scale’ principle (creating a subject, in this case a leg, with a defined and realistic scale by measuring the model used), and the three-point perspective principle (the use of three vanishing points to create illustration depth on a two-dimensional medium). The choice of the color palette for the illustrations was considered as highly important in a cross-cultural sense [30]. Using portraiture by Ghanaian artist Amoako Boafo as inspiration, color palettes of RGB values that matched African ethnicity were defined digitally [31], also taking into account reproduction considerations and the pathological presentation of disease [32]. Hence, a series of graphic communication principles were formalized to maintain a common underlying visual language. This included considerations of four main areas. First, the target audience, ranging from lay people, non-specialists and patients (who may have little or no health knowledge) to health providers (who may have a high level of specialist health knowledge). Second, the outlets, considered to be health education services and/or clinics, utilize printed educational materials (posters, leaflets, flyers, PowerPoints) as well as digital formats. Third, the skin tone, which should reflect the communities in which BU is most prevalent (West Africa) and be acceptable to them. Finally, the BU lesions themselves, which should provide a direct and immediate visual comparison of differences between the presentations, and as they appear on the skin surface whilst exemplifying individual disease characteristics and unique manifestations for easy viewer understanding.

Taking these into account, the illustrative style chosen was designed to be instructive, educational, culturally appropriate, clear and engaging. Resource availability (varying quality of printers) and cost considerations (such as those associated with print reproduction) were considered. To avoid merging and to retain clarity, areas of white space were left either around, or in between, individual illustrations and labelling was kept to a minimum. Two different Black skin tones were illustrated and tested. The lighter Black skin tone is more amenable to print reproduction with dark areas kept to a minimum and used next to lighter colours to help distinguish areas of tissue and pathology. The pathology of BU can be seen more clearly on the lighter skin tone. The darker Black skin tone was created again with white surrounding areas to retain clarity, and as it is closer to the native skin tone of the participant groups in this study.

**Fig 1:**
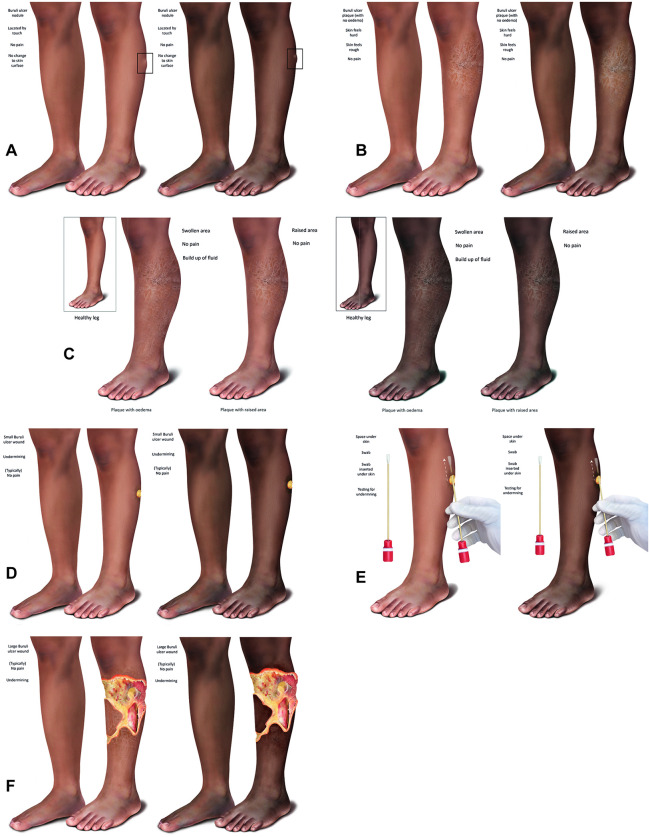
Medical illustrations of different clinical forms of Buruli ulcer shared with participants. The clinical presentation of the disease is shown on a leg in ¾ view, alongside a normal healthy leg for comparison. Different presentations of the disease are shown: nodule **(A)**, plaque without oedema **(B)**, plaque either with oedema or with no oedema and raised area **(C)**, small ulcer **(D)**, small ulcer with the undermined edge emphasized by the insertion of a cotton swab **(E)**, and large ulcer **(F)**. Each lesion is illustrated using two different Black skin tones (lighter Black, where the pathology is easier to see and darker Black that is closer to the skin tone of the participants’ communities). The illustrations were shared with participants as laminated individual high-resolution A4 prints.

The bodily location for the BU lesion was considered carefully to present an anatomical position that accurately reflects BU characteristics. Hence, a limb extremity was chosen, as this is the commonest site for BU lesions to be located [23,33]. To maintain consistency in the images, a single leg from the knee to the foot was illustrated and used repeatedly as the background. This provided a genuine comparison of BU presentations as they would appear on the human limb. The precise location and ¾ angle of the leg emerged from clinical feedback from a wide range of stakeholders at the WHO [28]. For the current study, a healthy leg was illustrated alongside, without detailed labelling, and instead with short clear statements to the side of the limbs. The darker and lighter Black skin tones that were shared with participants in this study are shown in Fig 1. The illustrations were individually printed at size A4 (8.27 inches by 11.69 inches) on glossy photographic paper using a high-resolution printer, then laminated to ensure all participants saw the same high-quality images.

### Study tool

The study employed a semi-structured focus group discussion guide (S1 Text) to collect participants’ opinions on current health communication tools and to ascertain views on the acceptability of using medical illustrations in them. The discussion guide was developed based on the tenets of two practical theories: the PICO framework [34,35] and the Theoretical Framework of Acceptability (TFA) [36,37].

#### The PICO framework.

This framework was adopted to guide the study and comprises 4 components: Population, Intervention, Comparison and Outcome [38]. This framework was adopted to develop the research tool and to promote the formulation of clear and focused research questions needed to generate evidence that has policy relevance.

In this study, the *Population* comprised categories of people selected to participate. These groups were considered important as they either conduct education activities (health providers and Community Based Surveillance Volunteers) or are often the targeted audience of such exercises (community members in BU endemic communities). The *intervention* in this study is the medical illustrations, described above. In the *Comparison* phase, we explored participants’ views on the research question: *Will illustrations produce less revulsion and be more acceptable as compared to photographs?* The *Outcome* constitutes the views and opinions of participants regarding the use of the illustrations, which we report in the results section.

#### Theoretical framework of acceptability (TFA).

We also used the Theoretical Framework of Acceptability (TFA) to guide the study [36]. This is a multi-faceted theory, adopted to explore the extent to which participants consider the illustrations appropriate, based on their anticipated cognitive and emotional response to them. Selected constructs from this framework, such as affective attitudes, perceived effectiveness, intervention coherence and self-efficacy, were included in the discussion guide to help investigate key dimensions of acceptability while also capturing the suggested changes considered necessary to ensure a wider acceptance of the illustrations. For instance, the affective attitudes were used to assess the positive and negative attitudes that participants had towards the visual modalities under study. To explore affective attitudes, participants were asked in the focus group tool about how they felt concerning the use of illustrations in place of real-life pictures in BU health messaging. The perceived effectiveness construct was also employed to investigate how participants believed that illustrations or real-time photographs would enhance their comprehension and information recall. Self-efficacy was used to determine whether participants felt confident in their ability to understand the information presented through the visual materials and to share that knowledge with others. This tenet also helped to investigate how participants perceive the visual modalities to influence their intention to seek care. Intervention coherence helped to assess how participants understood the benefits of the visual modalities under study. Other constructs of the TFA such as burden, ethicality and opportunity cost were not assessed in this study.

#### Study participants.

Participants included health providers [facility-based workers (nurses, physiotherapists and medical doctors involved in BU care) and Disease Control Officers (DCOs)], Community-Based Surveillance Volunteers (CBSVs) and community members from endemic communities. DCOs and CBSVs were included due to their involvement in BU community health programmes, such as case searches [39]. They provide health education using printed materials such as posters, leaflets, booklets, and other imagery to engage and inform affected communities. Some nurses are also involved in community health and undertake extensive outreach activities. Community members within the districts were also randomly selected and included. The different participant groups were considered necessary to explore the varied opinions from the different viewpoints of key stakeholders at the facility and community levels.

### Participant recruitment and study procedures

Health providers and CBSVs were recruited during a workshop to train community volunteers and key stakeholders for BU case search. Community members were recruited through a convenience sampling strategy. Recruitment was based on willingness, expressed in providing written informed consent. In the consenting process, the overall aim of the research was explained to participants. Members of the research team (YAA, RDT) clarified and addressed concerns. Participants who provided informed consent were recruited to join the study.

We conducted Focus Group Discussions (FGDs) over a three-month period in 2024 in the three purposively selected BU endemic districts in the Ashanti region of Ghana; the discussions were guided by trained experts led by RDT. We held discussions with 36 health providers, 31 CBSVs, and 49 community members from the selected districts. The participant number ranged from 6-12 per FGD. Data saturation guided the number of discussions held per participant category.

**Fig 2:**
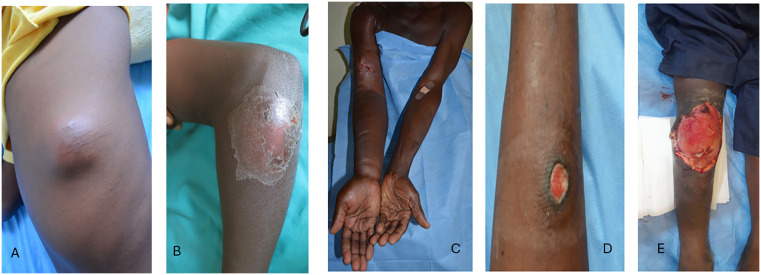
Real life photographs of different clinical forms of Buruli ulcer. Nodule **(A)**, Plaque **(B)**, Oedema (C) small ulcer (D) and large ulcer **(E)**.

During FGDs, copies of real-life photographs of BU (Fig 2) alongside medical illustrations (Fig 1) were shared with participants. For each stage of BU, photographs and illustrations were shown to participants by disease stage (nodule, plaque, oedema, ulcer). A focus group discussion guide (S1 Text) was used to comparatively assess participants’ views and opinions of the photographs and illustrations. All discussions were held at private venues, within health facilities and other private locations within communities in the study area.

All focus group discussions were recorded using an encrypted audio recorder to ensure the protection of the recording from any unauthorised user. The FGDs were conducted by a female researcher, RDT (MPhil) while other researchers [ST (BSc) and EOBB (MPhil)] recorded field notes. The FGD sessions were conducted in English or the local language, Twi, as appropriate. The FGD sessions lasted for 50–90 minutes.

### Data analysis

The interviews, conducted in the local language, Twi were translated and transcribed into English from the audio recordings by members of the study team (RDT, ST, EOBB, MG). The transcripts were then read, and sections of the audio recordings listened to for confirmation of the transcript. About one third of these transcripts were verified by an independent research scientist (AA, female) at KCCR who listened to the audio recording in the Twi language while reading the English transcription to establish the accuracy of the translations. All recorded interviews (including those in English) were transcribed verbatim and verified independently by 2 scientists (YAA, male and BA, female) to ensure quality control. Transcripts were assigned unique numbers to ensure the anonymity of the study participants. Using an initial selection of transcripts, RDT (female, MPhil) generated an initial coding framework. This was reviewed by YAA (MD, PhD) to ensure consistency and credibility. RDT coded all transcripts using MAXQDA software version 24. Data were analysed thematically. Coded transcripts and emerging thematic areas were then reviewed by YAA after which related topics were merged. From this process, 3 broad themes with various sub-themes emerged. The study’s findings have been reported in accordance with the Consolidated criteria for Reporting Qualitative Research (COREQ) checklist (S2 Text).

## Results

In all, eleven (11) FGDs; 4 with health providers, 3 with CBSVs, and 4 with community members were held.

### Socio-demographic profile of study participants

The study population consisted of health providers, community-based surveillance volunteers (CBSVs) and community members. The socio-demographic characteristics of participants are detailed in Table 1.

**Table 1:** Sociodemographic characteristics of participants.

Variables	Health providers, n = 36	Community-Based Surveillance Volunteers, n = 31	Community members, n = 49	All n = 116
Age group (years)				
≤24	0	1	3	4
≥25-64	36	21	41	98
≥65	0	9	5	14
Gender				
Male	19	25	23	67
Female	17	6	26	49
Educational status				
No formal education	0	8	14	22
Primary education	0	12	9	21
Secondary education	0	7	19	26
Tertiary	36	4	7	47
Marital status				
Married	28	26	37	91
Unmarried	8	5	12	25
Religion				
Christianity	19	15	36	70
Islam	10	11	10	31
Traditional	7	5	3	15
Occupation				
Employed	36	28	38	102
Unemployed	0	3	11	14
Ethnicity				
Akan	21	23	28	72
Mole-Dagbani	6	5	13	24
Others	4	3	8	15
Experience (years)				
1-5	7	7	N/A	14
6-10	18	0	N/A	18
> 10	11	24	N/A	35

In all, 36 health providers, comprising 16 Disease Control Officers, 1 Medical Doctor, 3 Physiotherapists, 1 Physician Assistant and 15 nurses, were involved in the study. 19 were male, while 17 were female. Their median (IQR) age was 44.5 (25–58) years. All 36 had attained tertiary education.

#### Health providers.

A total of 31 CBSVs were included in the study. 25 were males while 6 were females. Their median (IQR) age was 45.5 (24–72) years. A majority (28/31, 90%) are self-employed. Eight (8) of the volunteers had no education, while 12 had primary, 7 had secondary and 4 attained tertiary education. Many (n = 24/31; 77%) volunteers had volunteered beyond 10 years, while a small number of 7 had volunteered between 1–3 years.

#### CBSVs.

Of the 49 community members included in the study, 23 were males and 26 were females. Their median (IQR) age was 45.5 (24–67) years. Fourteen (14) of the community members had no education, while 9 had primary education, 19 had secondary education, and 7 had attained tertiary education.

#### Community members.

### Thematic areas

**Fig 3:**
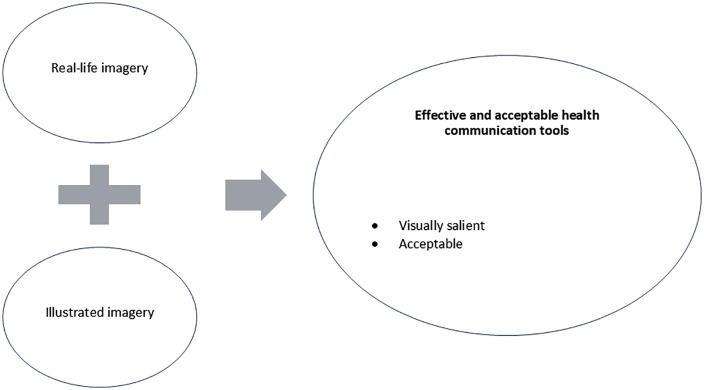
Schematic diagram of thematic areas.

Broady, three themes emerged: The current role of imagery, challenges associated with real life imagery such as photographs and the acceptability of medical illustrations in existing health communication tools. It was generally noted that, whereas the current real-life photographs in health communication materials play a critical role, they can be complemented with illustrated images for improved visual salience and acceptability (Fig 3).

#### Theme 1: The current role of imagery in health communication tools.

All participant categories noted that imagery of BU disease in forms such as printed posters, educational videos, PowerPoint presentations, brochures, booklets, leaflets, in addition to other educational content, constitute important health communication materials, crucial for community health education and sensitization programs and health-worker education on BU disease. However, the perceptions of usefulness and value differed according to participant category (Table 2).

**Table 2:** Matrix for theme 1 on the current role of imagery in health communication tools.

*Theme component*	*Health providers*	*Community-Based Surveillance Volunteers (CBSVs)*	*Community members*
*Usage in community education and sensitisation*	*Used posters, videos, etc in outreach and case search activities*	*Primarily tasked with displaying posters in vantage points in the community to stimulate discussions concerning BU*	*Encountered posters in markets, schools, health facilities and during outreach activities*
*Contribution to case identification*	*Considered imagery as useful for confirming BU signs and symptoms and used in consulting rooms for case identification*	*Highlighted that imagery assisted community members in reporting BU signs and symptoms to volunteers*	*Reported that they could better recognise disease signs and symptoms in themselves and others after viewing posters*
*Primary role and value of imagery*	*Imagery aid case identification, diagnosis and management, supporting clinical judgment and practices*	*Viewed imagery as crucial to community engagement and promoting the effectiveness of volunteer activities*	*Saw imagery as a key tool for awareness creation, contributing to improved understanding and knowledge about BU, prompting care seeking*

### Perspectives of Health providers

Health providers reported several ways in which imagery in health communication materials had been useful in their work. These include community outreach activities, disease identification and management.

1. *Community outreach activities:* Many health providers noted that posters and videos featuring images have been instrumental in community outreach activities such as education, awareness raising and screening exercises. The presence of such explicit images helps audiences in disease identification, contributing to improved case identification and encouraging appropriate care-seeking.

*‘It has helped in so many ways. With Buruli ulcer, if you don’t demonstrate any picture in the community, the case searching becomes difficult. Wherever the case may be, it helps for the case to be referred to the health facility’ [Health Providers FGD_ 002, P6].*

2. *Disease identification and management:* Health providers also reported that images in the form of printed posters pasted within their facilities and educational booklets in their consulting rooms helps them to identify the signs and symptoms of similar presented conditions, guiding them in their practice.

*‘What I have to say is that it helps us who work at the consulting room because when you get a case like that, it helps us to see how the condition is’ [Health Providers FGD_ 002, P2].*

### Perspectives of CBSVs

Volunteers mentioned that they normally receive health communication materials during workshops. They are mostly pasted on walls during awareness campaigns in public areas within their communities. Affected community members come to them, and they in turn refer them to the health facility, contributing to early care seeking;

*‘When we paste the pictures around and the people see it, and as we’ve talked to them about it if the person gets any skin condition, he will quickly seek medical attention and treatment. Because they’ve seen the terrible nature of the condition, they will also get close and ask questions about their conditions. As we talk to them, we direct suspected cases to the hospital. They get scared of it’. [Community Volunteers FGD_ 003 P4].*

Many volunteers reiterated that printed posters have helped them in community education and sensitization activities. Affected people, upon seeing posters, seek more information about the condition and this then allows volunteers to educate them and refer affected persons to health facilities;

*‘When I came here and was given the posters to paste in the community, one Monday morning, a woman came to me asking about a similar condition on her arm it was similar to the ones on the poster I pasted. When she showed it to us and we came here, it was positive. I think the poster has been very beneficial. There are some people that when you speak to the at the borehole centre, market or the community, they will report any form such conditions in their kids and relatives’. [Community Volunteers FGD_ 001 P6].*

### Perspectives of community members

Community members primarily valued imagery for raising awareness and shaping their care-seeking behaviours. They particularly reported that health personnel show them posters featuring the disease during active case search activities. Other times, they also see posters within their health facility premises, schools and other public areas like markets, contributing to awareness of the disease, and available care-seeking pathways. This also increases the likelihood of recommending appropriate care to other people;

*‘From the poster, I learned the signs and symptoms of the disease. And this helped me in creating awareness by informing people who show these signs to go to the hospital as soon as possible…..When I see someone showing the signs in the poster and the person thinks it is a normal wound or a boil, I inform them it is Buruli ulcer and that it is not something normal. I advise them to go to the hospital.’ [Community members FGD_ 003, P8].*

#### Theme 2: Challenges associated with the use of real-life imagery in health communication materials.

All participants affirmed that the existing health communication materials, which use real-life imagery, play a crucial role. However, reactions to them varied across the different participant groups (Table 3). Community members noted that photographs usually elicit strong emotional distress, and community volunteers noted that they may be emotionally distressing; highlighted observing concerning reactions from community members when using photographs to engage them. Health providers also affirmed that they may be emotionally distressing contributing to a feeling of revulsion and anxiety among viewers, especially people who are seeing the condition for the first time; however, they believed it to be important and an unavoidable component of sensitisation and awareness creation.

**Table 3:** Matrix for theme 2 on challenges associated with the use of real-life imagery in health communication materials.

*Theme component*	*Health providers*	*Community-Based Surveillance Volunteers (CBSVs)*	*Community members*
*Initial emotional reaction*	*Acknowledged that images are distressing, particularly for untrained, young and unexposed audiences*	*Noted that images are sometimes shocking, they also observed unfavourable emotional reactions among community members*	*Reported shock, fear and nausea, especially for those viewing for the first time*
*Behavioural responses to real-life imagery*	*Confirmed avoidant behaviours from community audiences*	*Reported avoidant behaviour of community members*	*Showed avoidant behaviours, including turning away, leaving sessions or removing posters*
*Perceived impact of images on engagement*	*Maintained that images may initially record unfavourable impacts but ultimately contribute to impactful engagement*	*Reported that some audiences become uninterested in health discussions after viewing materials*	*Some viewers disengaged completely and failed to absorb health messaging*

### Perspectives and experiences of health providers

Health providers such as Disease Control Officers, who are mostly engaged in active education and sensitization activities, including case searches, outreaches and community gatherings affirmed that they witnessed distressing reactions from community members during engagement activities with these materials;

*‘When you show a typical BU lesion which has ulcerated with slough to the community members, they bow their head and exclaim “Eiiii” because, in English, we say traumatophobia, the person is scared of blood, cut or open skin. Because the individual has not seen a condition like that, and seeing someone’s skin degrade it gives the individual a bad feeling, and as such bow their head. We have seen a lot. When we go into the community for surveillance work, they bow their head or close their eyes during the display of severe conditions [Health Providers FGD_ 001, P5].*

In one district, health providers shared an experience where community members removed a poster, complaining of the graphical content;

*‘…the next day, people had removed it. During an education session on the conditions, the people said they couldn’t look at it (posters of Buruli ulcer), and they don’t even want to see it. Because of that, they removed the poster the next day’ [Health Providers FGD_ 001, P8].*

*Moderating factors noted by health providers* Despite the general discomfort associated with the real-life images, we also noted that some factors predisposed the audience to different levels of stress and mental discomfort related to the images. These factors include: previous exposure, age, and the context within which the health communication materials are shown.

1. Previous Exposure: Previous exposure gained from past trainings or personal experiences related to the condition influenced the reactions expressed by participants. Clinically trained health providers, for instance, showed a more positive reaction to real-life images. They attributed this to their training and the nature of their work, providing care to patients with skin conditions.

*‘I am a typical example. During our school days, one of our tutors brought pictures and videos from Agroyesum (this was a typical Buruli ulcer endemic village). That was my first time seeing the disease. During the class lesson, he informed us that the video we are about to watch is on Buruli ulcer. Because we were new… (participant points at another participant and says: P3 here is my classmate… he then continues the narration) when he showed us the video, I do not think my mates who were able to watch it will be more than two…. We were first-year students …. I could not watch because the ulcers were big. (participant’s facial expression mirrored the shock he experienced) …It was serious. You could see someone with BU, which covers all the trunk and limbs. So, as a first-year student who had not even started with clinicals. I will never forget that moment. I recall those videos. It was very serious…. I couldn’t eat that very day…I was able to eat the next day. But as time went on, I realised I had come to the field, so it is normal, but with my first encounter, I couldn’t watch the screen.’ [Health Providers FGD_ 004, P7].*

2. *The age of the viewer:* Age was reported to be an important factor influencing participants’ reactions to real-life imagery in existing health communication tools. Younger people were reported to experience increased shock and revulsion as compared to older people;

*‘I was a little bit younger by then, and so I was very scared.’ [Health Providers FGD_ 004, P1].*

*‘Please, I’ve also seen one when I was younger. I panicked. I was scared when I saw it. I didn’t even want to look at it again because I couldn’t look at it again.’ [Health Providers FGD_ 004, P4].*

3. *Differences in context:* The contextual variation, such as public versus private spaces like the home setting, was also shown to affect the expression of emotional responses to health communication tools such as educational videos and printed materials featuring real-life imagery;

*‘When they (community members) are gathered at one place, the noise (expressions of aversion to the displayed condition) is much louder than when you visit them individually at their houses. Some people will choose to look at the posters, and others will tell you they can’t even eat their meat after seeing such photos…. When they are gathered, sometimes you may even, after informing them about the nature of the video, hear them still murmuring and making noises. However, when it is one-on-one in their houses, the way they shout and make noise is very minimal’ [Health Providers FGD_ 001, P12].*

### Current strategies adopted to manage revulsion associated with real-life images in health communication materials

Health providers involved in community work highlighted some mechanisms they use to manage the alarmed emotional response that real-life imageries normally elicit from their audiences. These include issuing viewer discretion, replacing the material with abstract descriptions and ignoring the reactions to the materials altogether.

1. *Viewer discretion:* Warnings are issued to audiences concerning the sensitive nature of the materials before they are shown. Although some people are still unable to watch, the majority can prepare mentally, thereby being less shocked and expressing more favourable reactions to the materials;

*‘From the beginning, before you start everything and show them the pictures, you will inform them of what they are going to see, like the way they do on TV. You prepare their minds on what they are about to see, and people who wouldn’t be able to watch will close their eyes… [Health Providers FGD_ 002, P5].*

*‘But we have observed that what normally ameliorates the situation is when you tell viewers that you’ll be showing them some pictures of this nature, which would be quite disturbing. Once they’ve anticipated it and you’ve sought their consent, and then you show what they are going to see, it kind of reduces their anxiety levels. They will not be so much disturbed but rather willing to point out people who have such conditions…. [Health Providers FGD_ 001, P2].*

2. *Providing an abstract description of the condition:* A few health providers reported that not everyone can view the materials containing real-life imagery, especially in the advanced forms of the disease. This is frequently seen during community outreach events where videos and photographs of affected people are shown. Despite the viewers’ discretion, some people are still unable to watch and look away. For such people, these health providers reported that they resort to describing the condition to and then following up with awareness raising messages to seek and refer others to care;

*‘… if they are not able to watch, I make the whole thing abstract for them’. [Health Providers FGD_ 003, P5].*

3. *Ignoring audience reactions:* Notably, many clinically trained health providers corroborated that although photography in health communication materials may disturb lay viewers, the images are necessary, as the associated shock will lead to more referrals of affected people for appropriate care. Many health providers also noted that the shock that viewers experience is an inevitable harm, necessary to incite fear of the disease, and thereby prompting early and appropriate care seeking;

*‘Someone viewing the real picture makes it more relatable. If the persons see such a thing on their skin or that of a different person, it will instil some fear in them to report or take action earlier. [Health Providers FGD_ 001, P5].*

Health providers reported that they often look past the sensitivity and discomfort their audience may experience when showing the materials to them. To them, this strategy proves to be effective since they find that community members, despite their initial discomfort, engaged with the outreach and contribute to improved referrals and care seeking;

‘*We forced to show it to them and talked to them…, so in the morning when they came back, and we enter the building, we got like about five people we were able to examine)’. [Health Providers FGD_ 003, P2].*

However, some community members and DCOs disagreed since viewers were revolted by the image and lose interest in the health communication materials altogether, thereby not accessing the information that these materials were designed to give;

*‘I remember that health workers came to show us videos for us to see how Buruli ulcer is like. But the problem was that we were there that night, many people could not watch it’ [Community members FGD_ 004, P7].*

*‘When the video starts to play and the people see it, they all bow their heads and murmur. The people were not encouraged to watch the video we were showing them. They rather bowed their heads, and some said they wouldn’t be able to eat if they looked at the videos. Others said the images would unsettle them mentally. The people were running away from the video. That is what I experienced during that time.’ [Health providers FGD_ 001, P12].*

#### Theme 3: Acceptability of medical illustrations.

We explored the acceptability of medical illustrations and found that they are likely to play a complementary role in health communication. Images currently play a critical role in health communication and while photographs are well-established, medical illustrations may have a more positive reaction from community members and are likely to work better under certain circumstances depending on factors such as the setting where the material is used, the audience type, and the severity of the condition being depicted (Table 4).

**Table 4:** Matrix for theme 3 on Acceptability of medical illustrations.

*Theme component*	*Health providers*	*Community-Based Surveillance Volunteers (CBSVs)*	*Community members*
*Overall acceptability*	*Illustrations are acceptable and could be particularly helpful for sensitive audiences or when illustrating more advanced lesions*	*Illustrations are acceptable and are appropriate engagement tools*	*Illustrations are emotionally comfortable and acceptable*
*Preferred setting*	*Illustrations are suitable for schools, churches and community display settings*	*Illustrations are good for community meetings and house-to-house meetings*	*Illustrations are ideal for community display settings*
*Skin tone*	*Preferred lighter Black skin tones for clinical clarity*	*Indicated no strong preference but support clarity*	*Mixed views, however majority preferred lighter Black skin tones as more visually appealing*

### Perspectives of health providers

Health providers considered images to be important for clinical recognition and clinical accuracy and are particularly useful in health facilities and in one-on-one meetings. However, some noted that, illustrations may be considered for public and non-clinical settings. Disease Control Officers noted that, using illustrations in schools, churches and community gatherings may have some benefits in reducing emotional stress which may be associated with the real images;

*‘The real pictures can be very bad. Everyone has a way of reacting to what they see. So, for me, if it’s going to be pasted in schools and communities, we should use the illustrations… and also, the real pictures have to be with us so we can use them from house to house for our training and education. Let’s paste the illustrations in the community. I think that will be helpful’. [Health Providers FGD_ 002, P6].*

Some health providers also associated acceptability with disease severity. They noted that, whereas photographs are suitable for representing the less severe forms of the disease, illustrations may be more acceptable for representing the more advanced forms of the disease, such as large ulcers. They observe that this will contribute to increasing accessibility since the advanced stages of the condition usually receive a negative emotional reaction from viewers;

*‘In my opinion, I suggest that when using posters, since the progression of the disease appears in stages, we should start with real images during the education sessions. However, when showing more severe cases, we should switch to illustrated images, especially during large group meetings, it would be better to use illustrations for the more advanced stages to avoid discomfort’. [Health Providers FGD_ 003, P8].*

It was observed further that the audience type is an important factor to consider in determining acceptability of illustrations. DCOs argued that illustrations can be used for a more sensitive audience, whereas the photographs can be used for audiences that are comfortable with it. Participants reiterated that this would help in more effective community messaging about the disease;

*‘What I think is that in a typical case searching, you can’t have a one-way approach. This is because everyone receiving information or looking at a picture is affected differently depending on their subjective understanding. We must understand that in as much as we use the real pictures, not everyone can accept or tolerate looking at the real photos closely. For some people, it might traumatise them psychologically. In certain instances, the illustrated ones will be more acceptable to people who can’t tolerate the pictures. So, there’s no one way approach that we can use in identifying cases in a typical case searching setting. So, if you have varying methods and approaches, and they all lead to the ultimate goal of getting more cases. I think that will be very good rather than a one-way approach….’. [Health Providers FGD_ 001, P2].*

### Perspectives of community volunteers

Community volunteers viewed acceptability through an engagement-focused lens. They iterated that illustrations could help them to better navigate diverse reactions from audiences. Volunteers also agreed with the combined approach highlighted by health providers; using illustrations for sensitive audiences and real- life images for more receptive audiences;

*‘…I will say that getting the two or getting the illustration to supplement the real one will be the best. Because sometimes in the community, reactions on people’s faces aren’t appealing when they see the real images. The illustrations complementing the real will be better. And we shouldn’t be relying on only one. We should always go with the two when reaching out to the community. So that whichever becomes handy, you can use it’. [Community Volunteers FGD_ 001, P6].*

### Perspectives of community members

Community members primarily had a high acceptability for illustrations based on emotional comfort. They affirmed that although educational materials with real-life images are good at providing education related to the condition, it also has some negative effects on their emotional states after watching, an illustration will therefore be helpful in bridging this gap;

*‘What I think is that we should include “this one” (referring to illustration) …I think that the illustration is best. It shows more details of a disease that can harm people, but the person watching is also okay and does not feel sad to watch it’ [Community members FGD_ 002, P2].*

*‘Anything that looks like a drawn figure is fine but when real pictures like wounds are placed on the poster, that’s when we get reactions. As for me, the drawing is better [Community members FGD_ 004, P6].*

## Recommended choices to optimise the effectiveness of medical illustrations

Although many participants expressed that medical illustrations would be of benefit to health communication materials, we further explored opportunities for improving the illustrations for enhanced acceptability across all groups. Some suggestions were raised on the colour palette, the size of the illustrated images, the background of illustrations, choice of body parts and protection of the materials from harsh environmental conditions, which will be considered in turn;

### Colour

To explore participants’ thoughts on the preferred complexion of the illustrated images, we presented illustrations of the differing clinical presentations of BU, each with two skin tones: a darker and a lighter black skin tone. The clinical view was that the lighter skin tone would allow for more accurate illustration of the changes to skin during BU, and indeed in the illustration development process the skin tone was lightened in response to clinician feedback. We wanted to understand if this resonated with people from affected communities.

A few participants preferred the darker skin tone, because BU mostly affects people from West Africa who have a darker skin tone and so illustrations should be made using darker colour pallets to resonate with reality;

*‘We are Africans and most of us are blacks, so let’s use the black’. [Community members FGD_ 002, P3].*

However, like the clinicians, the majority of participants preferred the fairer-toned illustrations. They acknowledged that, the diseases being a tropical disease should be illustrated with an ethnically appropriate skin tone; however, this should be fair enough for improved visual salience. They maintained that the fairer tone, while still a tropical colour shade, was clear and should be able to show key details of the condition being illustrated;

*‘I think we should use the fair one because, unlike the dark, you can see the condition even at a distance. So, let’s use the fair one’. [Community Members FGD_ 003, P6]*

### Size

One of the central suggestions, participants conveyed was on the size of the medical illustrations in health communication materials. They noted that a bigger size is more likely to be effective, especially if they are in the printed form, like posters. This way, posters pasted on walls around communities will stand out and are more likely to garner attention as compared to those with smaller illustrations;

*‘…you should enlarge the images on it. Make it bigger. For the small ones, not everyone has good eyes to see them. It will be better if it’s enlarged. [Health Providers FGD_ 004, P7].*

*‘For the size, let’s enlarge it because people don’t pay attention to these smaller ones. Making it big will draw their attention to the urgency of the condition. They will see things very well. So, let’s enlarge the posters and the illustrations on them as well. [Community Volunteers FGD_ 003, P2].*

Other minor recommended changes, that were made included presenting illustrated images using familiar backgrounds and the representation of typically affected body parts to portray the illustrations.

### Background

One participant noted that the presented medical illustrations have a white background, making the condition look less real. As such, some familiar environmental contexts, such as a hospital or community setting, could be provided as the background of the illustrated images to mimic real-life situations and make the illustrations more relatable, as well as promoting visual salience;

*‘I think when you look at the two real pictures, the environment even shows that it’s humans we’re talking about…with the illustrations, if we can get something catchy for us to relate with it, that’ll be fine… For example, when you look at the real pictures, you get to see that the person is in a health facility, and so on….’. [Health Providers FGD_ 001, P8].*

### Use typical/differing body parts

Another participant expressed that the typically affected body parts aside the limbs should be mostly represented in illustrations to stay consistent with reality.

*‘we must use different body parts…the parts that the disease normally affects like the hands and legs, so people are able to relate to it’ [Community Volunteers FGD_ 002, P5]*

### Protection of printed materials against harsh environmental conditions

Participants, specifically, CBSVs also shared a key challenge they face with the current materials. Particularly with printed materials for display, such as posters that are pasted within health facilities, schools, markets and other community public viewing areas. These materials do not last due to harsh environmental conditions, such as winds, rain and the sun. They are therefore defaced or succumb to wear and tear within a short time. To remedy this, some suggestions were made like pasting these posters in strategic locations where they are not constantly exposed to rain and sun.

*‘It’s been a long since we started attending workshops and training. They mostly give us the posters. Sometimes when you paste it, environmental conditions like wind and rain remove it’. [Community Volunteers FGD_ 001, P10].*

*‘…. Even if it’s only one and it’s pasted at a good location, it will be better than the posters that are affected by our weather conditions. [Community Volunteers FGD_ 001, P6].*

## Discussion

This study investigated the acceptability of photographs and medical illustrations in Buruli ulcer health communication among community volunteers, community members and facility health workers and from communities in endemic districts of Ghana. It was found that, images play a crucial role in community education and sensitization, awareness creation and assists health providers in diagnosis and treatment. However, photographs are sometimes emotionally disturbing, especially for community audiences and first-time viewers. Medical illustrations were found to be acceptable and likely to be an important complement to existing health communication materials. The potential contribution of illustrations varied by context, audience type, disease severity and age of the viewer.

The nature of images currently included in these health communication materials focuses on real-life photographic images, often taken in clinical settings. In line with our hypothesis, participants confirmed that, despite the usefulness of having imagery, the photographs are associated with fear and revulsion. We found that the existing health communication materials contribute to an improvement in community awareness, similar to previous studies, which acknowledged that health communication materials are widely used not only to increase awareness and knowledge but also to contribute to a change in beliefs and attitudes [40–42]. These forms of campaigns have been used extensively in many conditions, including cancer, heart disease, stroke, HIV/AIDS, and obesity [43].

In our study, health providers and volunteers affirm that they often ‘receive’ these materials from the government and other stakeholders, and their role involves using these materials during health education activities. However, little enquiry is conducted to find how acceptable these may be for communities and the recipients of these materials. This is similar to that previously reported by Zingue et al, where communities are noted to frequently play little to no role other than receiving resources and tools, which are developed utilising top-down approaches [44]. However, this seems to be a long-standing issue as similar reports are given in other conditions. For instance in skin cancer, it was reported that studies often fail to discuss theories of visual communication, and principles such as visual persuasion, technical design characteristics and visual salience [45]. Therefore, there seems to be huge scope for co-creation of educational and public health materials with the affected communities.

Since medical illustrations have not been deployed for health communication of BU or other skin NTDs in these settings in the past, the illustrations of BU went through extensive feedback to ensure clinical accuracy and representation of the diseases. Our data showed strong support for the incorporation of such medical illustrations in BU health education materials. Health providers, volunteers and community members corroborated that the illustrations are a good fit, especially to depict BU in the advanced stages, as the real images of this stage often receive a negative reaction. Additionally, illustrations will be better accepted in settings such as schools and religious gatherings because they are visually appealing, as compared to the use of real-life imagery in such settings. This finding ties in with other research in skin cancer and concussion, which report that illustrations alongside other interactive content, like infographics (that were deliberately excluded from the illustrations shared with the current participants in order to focus on the illustrations as opposed to associated elements) are likely to be more effective [46]. Illustrated communication materials were noted to contribute to a reduction in the anxiety levels and increase patient satisfaction and comfort levels among cardiac surgery patients [19].

In our study, health providers noted that the use of fear appeals in real-life imagery, featuring the disease in late stages in health communication materials, is effective since this arouses an emotional response and promotes care seeking and referrals. However, the use of material as such scare tactics in health promotion may not only have ethical, moral and political implications, but even if not deliberate may also potentially contribute to anxiety and denial since the advanced stages of the condition used may not be what people normally see in their communities [47–49]. The anxiety associated with the material makes the campaign unattractive and eventually causes more harm than good [48,50]. Additionally, the use of such materials also poses a challenge to human dignity, as well as perpetuating stigma and other forms of marginalisation for affected people within endemic communities [43].

## Study strengths and limitations

This study fills an important gap in the literature as the subject under review has remained unexplored in Buruli ulcer health communication. By focusing on medical illustrations, this study contributes novel evidence to inform communication strategies for awareness creation in BU and other skin NTDs by providing contextually grounded insights into the acceptability of medical illustrations for BU health communication. The study also involved different categories of participants, including health providers, community health volunteers and community members, thereby providing an opportunity to triangulate perspectives to comparatively explore professional as well as lay viewpoints and experiences concerning the use of differing tools in BU health education. Yet the study has some limitations. First the use of a qualitative design, which although provides rich data, is inherently contextual and therefore the findings may not be generalizable to other contexts. Also, the respondents in the focus group discussions may have proffered socially desirable answers. Participants, particularly health providers and CBSVs, may have framed their responses to align with perceived desirable professional expectations and not according to their realities. Also, group dynamics may have influenced individual dissenting opinions on the subject matter. Further, health providers and CBSVs may have reported on observed community acceptability based on their professional experiences and not on their personal views on acceptability.

## Conclusion

Medical illustrations have a potentially important role to play in BU health messaging in West Africa. We found that, they are well suited to depict BU, particularly in the late stages to produce health communication materials that do not invoke negative emotional responses. Furthermore, medical illustrations are likely to work well in health communication materials used in settings such as schools, churches and within other community areas like markets, where the audience is likely to be lay or younger people. However, health communicators working on skin NTDs must work towards situating materials within the appropriate social and cultural contexts, as these are necessary to promote the effectiveness of the material and the uptake of the messaging. Our long-term aim is to increase early detection of BU, and this work has supported that medical illustrations can be an important part of that goal and provided some framework for how this may be best achieved. As medical illustrations become more widely used, and potentially also in training material for medical professionals, a future goal would be to understand their true impact on detection rates.

## Supporting information

S1 Text Topic guide for Focus Group Discussion. (PDF)

S2 Text Consolidated Criteria for Reporting Qualitative Research (COREQ) checklist. (PDF)
