# Supplementary material for: Assessing the acceptability of photographs and medical illustrations in Buruli ulcer health communication among health providers, community volunteers and community members in endemic districts of Ghana
Source: PLoS Negl Trop Dis. 2026 Apr 22;20(4):e0014275. doi: 10.1371/journal.pntd.0014275 (PMC13128114; doi:10.1371/journal.pntd.0014275)
Supplement: S1 Text — (PDF) [file pntd.0014275.s001.pdf]

Topic Guide – Focus Group Discussion (FGD)

Target group:

- Community members, health workers, etc.

Key research questions follow-up:

1. *How* **acceptable** *are the medical illustrations in BU health communication, compared to real-time photographs?* ***(visual appeal, emotional response, validity, social acceptability and cultural appropriateness).***

   1a. How does the use of the medical illustrations/real-time photographs promote **knowledge sharing** about BU?

   1b. Does the use of the medical illustrations have an effect on **behaviour and health-related practices**? ***(changes in illness beliefs, health-seeking and adherence),*** compared to real-time photographs?

Note for each FGD:

- Interview date
- Venue
- Group Code
- FGD type
- Number of participants
- Socio-demographic information (*separately for each participant*)

**Domain**	**Questions**
**Acceptability of the medical illustrations in health communication**<br><br>- *Emotional response*<br>- *Social validity*<br>- *Cultural appropriateness*<br>- *Visual appeal*<br>- *Context specificity*	o Can you discuss the roles of illustration and real-time photos in accurate presentation and diagnosis *(any differences?)*<br><br>o How do you feel about the use of illustrations in place of real-time pictures in health messaging ***(Probe about B*****U presentation, diagnosis, Treatment, etc as shown in the leaflets)**<br><br>o What are your perceptions on the use of medical illustrations in treatment-related messaging in contrast to the use of real-time photographs?<br><br>o Which types of settings/locations and for which groups of persons would you recommend illustrations or real-time pictures for health messaging?
**Role of Medical Illustration in Knowledge Sharing**<br><br>- *Attention*<br>- *Understanding/ comprehension*<br>- *Recall*	o Can you share what you learnt about BU in the leaflets that were shared with you? ***(Probe to find knowledge about BU nodule, plaque, ulcer and sample taking)***<br><br>o Would you consider the content of the document as easy to identify, understand and comprehend?<br><br>o How confident are you that you can explain these to another person? ***(Probe to test self-efficacy of participants to pass on the acquired knowledge related to identifying BU nodule, plaque, ulcer and sample taking).***<br><br>o What would you consider to be **attention-grabbing** from the document? Why?

	○ What changes would you propose to the document to make it more comprehensive and easier to recall for other people?<br>○ Are there specific changes you would recommend for some categories of people? *-community members? Community volunteers? Health workers? Affected persons?*
**Effect of the medical illustrations on behaviour and healthcare practices**<br>- *Illness beliefs*<br>- *Health seeking*<br>- *Adherence to care*	○ In which ways has the document affected your beliefs and perceptions about BU? **(Probe: practical examples of changes associated with beliefs related to BU aetiology, presentation, etc)**
